# Supplementary material for: Development of LAMP assay for early detection of Yersinia ruckeri in aquaculture
Source: PeerJ. 2025 Feb 25;13:e19015. doi: 10.7717/peerj.19015 (PMC11869897; doi:10.7717/peerj.19015)
Supplement: Supplemental Information 1 — The underlined regions represent the complementary to 5′–3′ sequence. [file peerj-13-19015-s001.docx]

Supplemental Table 1: Conventional polymerase chain reaction (cPCR) primers and conditions used throughout the current research. The underlined regions represent the complementary to 5′–3′ sequence.

| **Test** | **Primers** | **Sequence (5′–3′)** | **Length (bp)** | **Final concentration** | **PCR conditions** | **Amplicon size (bp)** | **Source** |
| --- | --- | --- | --- | --- | --- | --- | --- |
| **Colony PCR** | M13 Forward | GTAAAACGACGGCCAG | 16 | 0.4 µM | 94°C 10', 35x (94°C 1', 50°C 1', 72°C 110''), 72°C 10' | *Y. ruckeri* (830), *Y. rohedi* (843) and *Y. frederiksenii* (837) | PCR™II-Blunt-TOPO® |
|  | M13 Reverse | CAGGAAACAGCTATGAC | 17 | 0.4 µM |  |  |  |
| ***Yersinia ruckeri* conventional PCR** | YR glnA F | TCCAGCACCAAATACGAAGG | 20 | 0.4 µM | 94°C 10', 40x (94°C 1', 64°C 30'', 72°C 30''), 72°C 10' | 109 | (Keeling et al., 2012) |
|  | YR glnA R | ACATGGCAGAACGCAGATC | 19 | 0.4 µM |  |  |  |
| **Universal bacterial assay** | Fn3 | CAGGATTAGATACCCTGGTAGTCC | 24 | 0.1 µM | 95°C 3', 33x (94°C 90'', 52°C 1', 72°C 105''), 72°C 3' | Various bands size | (Barghouthi, 2011) |
|  | F4 | CCGCCTGGGGAGTACG | 16 | 0.1 µM |  |  |  |
|  | Fn5 | ACTCCTACGGGAGGCAGCAG | 20 | 0.1 µM |  |  |  |
|  | Fn6 | CCAGCAGCCGCGGTAATAC | 19 | 0.1 µM |  |  |  |
|  | Rn1 | GGCTACCTTGTTACGACTTC | 20 | 0.1 µM |  |  |  |
|  | Rn2 | TGACGGGCGGTGTGTACAAG | 20 | 0.1 µM |  |  |  |
|  | Rn3 | GGCGTGGACTACCAGGGTATC | 21 | 0.1 µM |  |  |  |


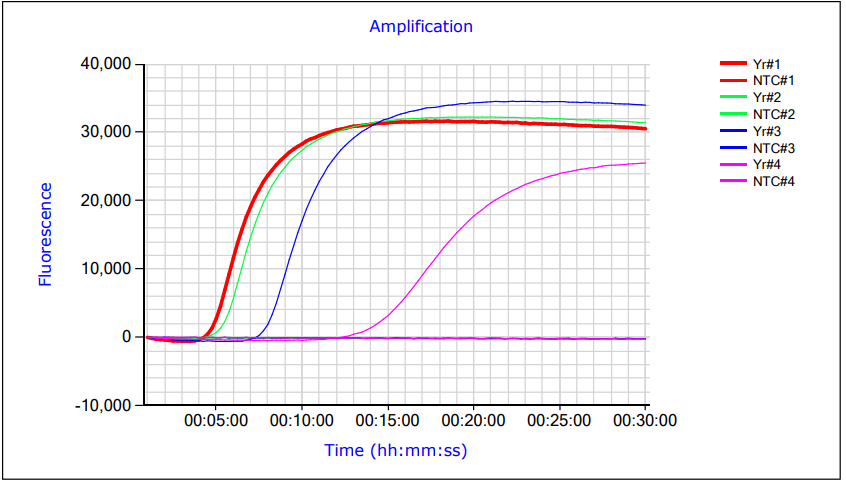


Supplemental Figure 1: Primer sets screening. The fluorescence curves generated from the amplification of the glutamine synthetase (*glnA)* gene of *Yersinia ruckeri* using four different primer sets, Yr#1, Yr#2, Yr#3 and Yr#4. Template was replaced with TE buffer in no template control (NTC). The thickened line represents Yr#1 which recorded the lowest time to positive.

**
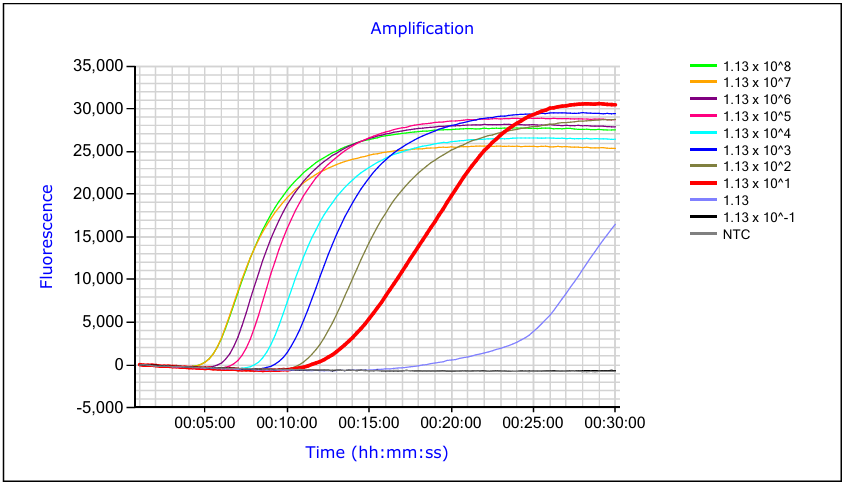

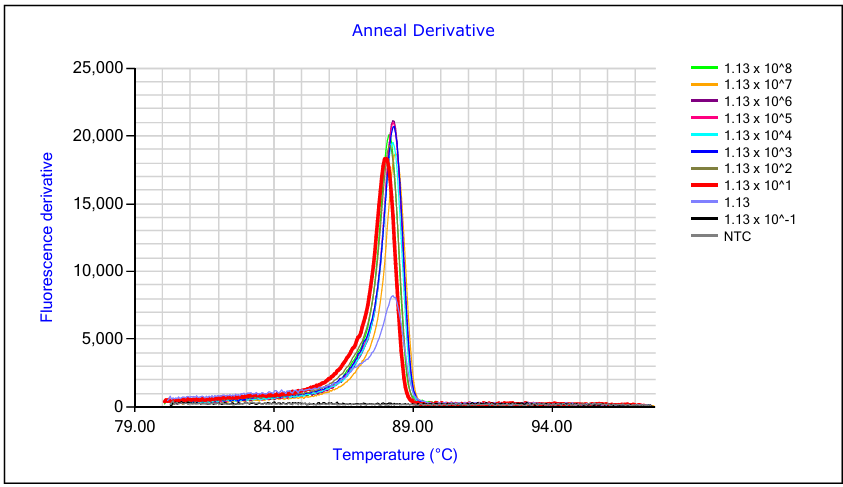
**

B

A

Supplemental Figure 2**:** *Yersinia ruckeri* (*Yr-*LAMP) Limit of detection (LOD). Ten-fold serial dilutions of plasmid containing the target gene (*pYr*) starting from 0.5 ng/µl, equivalent to 1.13 x 10^8^ copies/µl were tested by the optimized *Yr*-LAMP assay. The results were recorded as (A) amplification curves and (B) melting temperature to confirm the amplicons. The thickened amplification curve illustrates the selected LOD for the assay.

**0.5 ng/µL**

**Ladder**

**0.5x10^-1^ ng/µL**

**0.5x10^-2^ ng/µL**

**0.5x10^-3^ ng/µL**

**0.5x10^-4^ ng/µL**

**0.5x10^-5^ ng/µL**

**0.5x10^-6^ ng/µL**

**0.5x10^-7^ ng/µL**

**0.5x10^-8^ ng/µL**

**0.5x10^-9^ ng/µL**

**Negative control**

**bp**

**200 –**

**100 –**


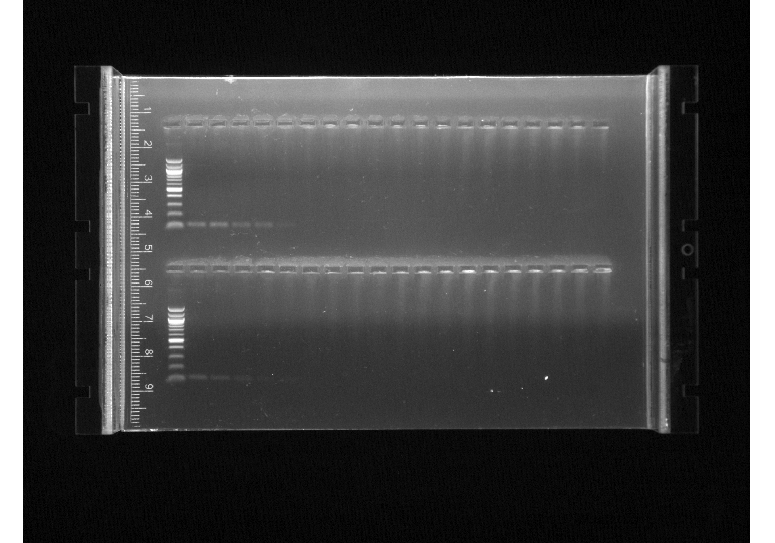


Supplemental Figure 3**:** Limit of detection of *Yersinia ruckeri* amplification using conventional polymerase chain reaction (cPCR). Ten-fold serial dilutions of plasmid containing the target gene (*pYr*) starting from 0.5 ng/µl, equivalent to 1.13 x 10^8^ copies/µl were tested by cPCR. The expected band size of positive amplification is 109 bp (red arrow) of glutamine synthetase (*glnA*) gene specific to *Yersinia ruckeri*. 5 µl of the cPCR product was loaded to 1.5% (w/v) agarose gel with 0.2 μg/mL Ethidium Bromide and resolved for 35 minutes at 100 volts. TE buffer replaced template in negative control samples.


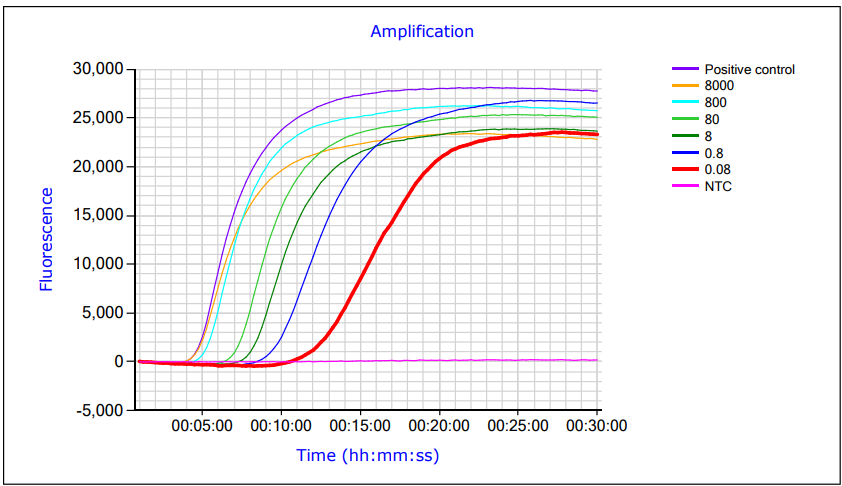

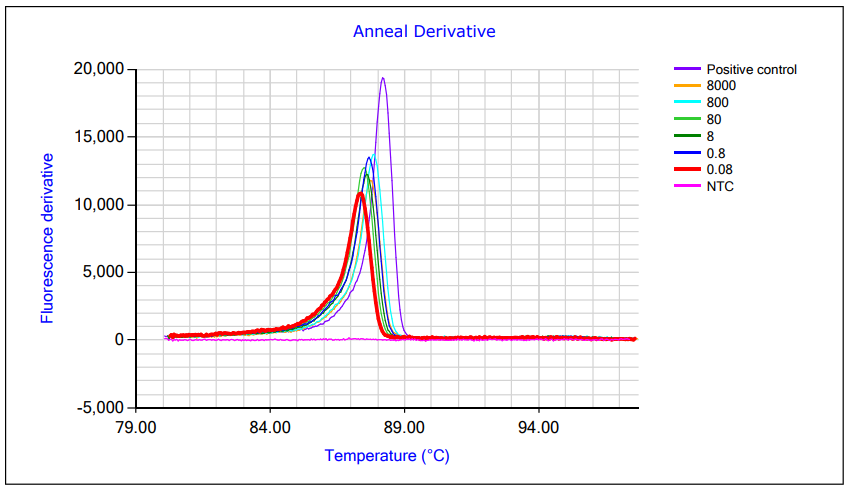


B

A

Supplemental Figure 4**:** Yr-LAMP assay for environmental water testing. Serial dilution of *Escherichia coli* bacterial cells containing *pYr* was spiked in environmental seawater to make a total volume of 50 ml. The final bacterial count in each solution ranged from 8000 cells/µl to 0.08 cells/µl. Filtered water extract was mixed with KOH. Direct 5 µl of the mix was tested by Yr-LAMP assay to evaluate the sensitivity of potential in-field filtration and extraction sampling. Data was reported as (A) amplification curve and (B) melting temperature for confirming the amplification. The thickened line indicates the lowest bacterial count detected by Yr-LAMP

**Positive control**

**Ladder**

**8000/µl**

**800/µl**

**80/µl**

**8/µl**

**0.8/µl**

**0.08/µl**

**Negative control**

**bp**

**200 –**

**100 –**


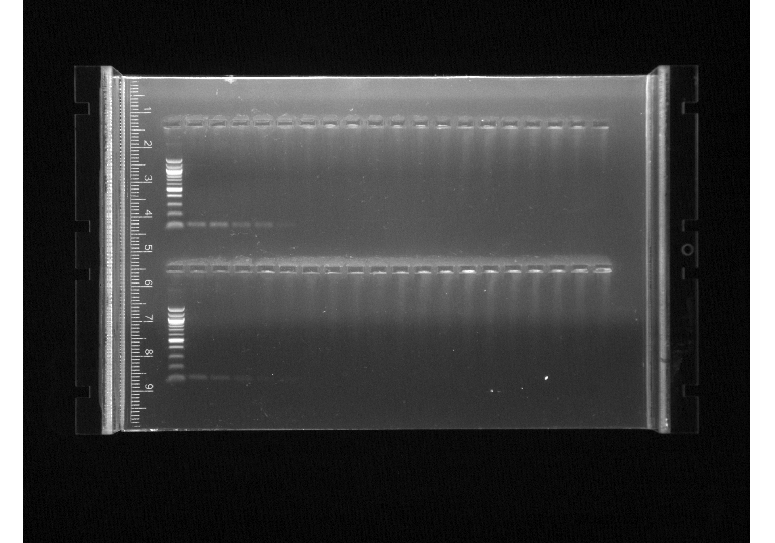


Supplemental figure 5**:** Sensitivity of filtration and in-field extraction of *Yersinia ruckeri* using conventional polymerase chain reaction (cPCR) amplification. Serial dilution of *Escherichia coli* bacterial cells containing plasmid (*pYr*) harbouring the gene of interest was spiked in environmental seawater to make a total volume of 50 ml. The final bacterial count in each solution ranged from 8000 cells/µl to 0.08 cells/µl. Filtered water extract was mixed with KOH. Direct 5 µl of the mix was tested by cPCR targeting glutamine synthetase of *Yersinia ruckeri*. The red arrow represents the expected 109 bp band size of successful amplification.

Barghouthi, S. A. (2011). A universal method for the identification of bacteria based on general PCR primers. *Indian Journal of Microbiology*, *51*(4), 430-444. <https://doi.org/https://doi.org/10.1007/s12088-011-0122-5>

Keeling, S., Johnston, C., Wallis, R., Brosnahan, C., Gudkovs, N., & McDonald, W. (2012). Development and validation of real‐time PCR for the detection of Yersinia ruckeri. *Journal of Fish Diseases*, *35*(2), 119-125. <https://doi.org/https://doi.org/10.1111/j.1365-2761.2011.01327.x>

**References:**
